# Notes of Surgical Cases

**Published:** 1862-06

**Authors:** J. F. Miner


BUFFALO

### Medical and Surgical Journal

AND REPORTER.

VOL. I. JUNE, 1862. NO. 11

### ORIGINAL COMMUNICATIONS.

ART. I.—NOTES OF SURGICAL CASES.—*Traumatic Aneurism; Club Foot; by* J. F. MINER.

*Traumatic Aneurism of the Brachial Artery.*—Frank Christ, German; healthy and robust laborer; was injured about March 1st, 1862, while at labor as a blacksmith. He supposed that a piece of steel striking his arm had penetrated or ruptured the vessels. Blood spirted from the wound very freely, and Dr. D. of Homeopathic persuasion, was called to dress the arm; "bandages were applied and *medicines given.*" A tumor soon commenced to form near the bend of the elbow, and continued gradually to increase, though a *solution of borax was applied, and a few drops directed daily, internally.* At the end of eight weeks the patient was advised to go to his work, with the assurance that it would be "*better for him.*" He now presented himself to Dr. G. N. Burwell for examination and advice, who, after discovering the nature, and extent of the disease, directed him to me for operation.

The tumor presented near the bend of the elbow, upon the forearm, and was about the size of a man's closed hand. It was a hard, elastic, pulsating tumor, and upon placing the ear upon it, the peculiar *bruit* of aneurism was distinct and unmistakable.

*April* 29, 1862, assisted by Drs. Lothrop and Eastman, with medical students, (Dr. Burwell being unable to be present,) the brachial artery was first ligated; in this respect not following the present fashion in traumatic aneurism, of opening the sac, and tying the artery where it enters the tumor. The veins were found distended and enlarged, in some instances partially obstructed. After tying the artery all pulsation and murmur ceased. The sac was now freely opened, and great quantities of coagulated blood, fibrinous deposit, and the partially formed walls of the tumor were removed. The hæmorrhage was profuse, both arterial and veinous; the collateral circulation supplied the artery, and blood poured from the vessel at the base of the tumor in astonishing rapidity; in all, many ligatures were necessary to control the hæmorrhage. The tumor was formed external to the affected artery, and without aid from its coats, which were altogether excluded from its composition, and constituted what has been denominated, diffuse, or false aneurism. The coats of the vessel had been ruptured, giving rise to the hæmorrhage at the time of injury, but pressure readily controlled it. Blood was afterwards effused into the neighboring cellular tissue. The sac commenced in this manner was early strengthened by effusion of plastic matter, which was still pliable and easily broken down and removed, though it had acquired a considerable thickness.

At the base of the tumor there was found two pieces of bone, or one piece, which in removing, was broken. They appeared like scales from outer portion of bone. The question of origin is not very satisfactorily answered. The steel penetrating might have caused exfoliation of the bone, and thus in this location, bone substance been found. It has been suggested that the obstructed circulation in the vessel had favored the osseous deposit, and that the bone had formed in the vessel. It did not look to me like the osseous deposit sometimes found in blood vessels.—Was it the substance driven from without, and producing the injury? It would seem impossible that so light a substance could be made to penetrate to that depth; and not the slightest support can be obtained from the circumstances at the time of the injury. The question of its origin and history, is not very satisfactorily answered. I shall be willing to allow any one to explain it in his own way.

The circulation in the arm was considerably deficient after the operation, and the third day the integument which had rested over the tumor was found completely sphacelated, the surface around looking blue, and the skin separated or loose upon it. It was placed in warm water dressings, and no further trouble occasioned. The wound made in tying the brachial artery healed by first intention; and the one upon the forearm was completely cicatrized in about four weeks from the time of operation.

There are many points which might be discussed in the operation above described, if that was the design of this report, and space would allow. The necessity of opening the sac, rather than allow the process of consolidation and absorption to complete the cure, is one of the practical questions which will be proposed, and no doubt differences of opinion will be entertained. The next topic suggested is, why ligature the artery at a distance from the tumor rather than follow the more recent practice of tying the artery near where it enters it? To these two questions I will briefly reply by way of explanation for my choice of operation. The tumor had attained considerable size for this location; the vessels were evidently quite numerous and greatly enlarged; consequently the danger of recurrence was the principal motive for opening the sac. The belief that it was equally safe, a more certain and expeditious mode of removing the complaint. also operated in the decision. It was not very certain which artery was involved, brachial, radial or ulner; the exact place of rupture could not be definitely ascertained; and the possible difficulty in finding and safely ligating the vessel or vessels supplying the tumor, made us regard it more prudent and convenient to ligate the brachial artery. This opinion was confirmed by noticing in a standard author's account of the disease, the following:—"The operation of opening the sac, turning out its contents, and ligaturing the vessels supplying it, is, under any circumstances, a procedure fraught with the greatest danger to the patient, and full of difficulty to the surgeon, even when he knows in what situation to find the bleeding vessel. How much greater then must the difficulty be when he is in uncertainty as to the point where the artery enters the sac, or the number of vessels by which it is supplied?"

This remark of Erichsen, seems to magnify the dangers and difficulties of the operation. The results in the case cited justify the procedure in this instance, though other modes of operation might, and probably would have been attended by equally happy results.

*Club Foot.*—March 5th, 1862, visited the child of C. W., three days old. Found both feet turned inward in the most distressing manner, the great toe resting upon the inside of the leg, by the side of the tibia, constituting the most common form of club foot, usually denominated *varus*, but existing in most uncommon, and unheard of degree.

The tendo Achillis was first divided. The tendon of the anterior tibial muscle was found forming a prominent tense cord; was much nearer the internal malleolus than in the natural state; this was also divided, when the foot could be brought down to a natural position, without much difficulty. The foot was retained in position by adhesive plaster, and laid upon a splint from those supplied by Welch for the fore-arm; in other words a splint accurately fitted to the leg, was well padded and applied, so that the foot was placed and retained in proper position. It will be noticed that the child was three days old, and that but one foot was operated upon; not the slightest disturbance in the general health was produced; the child remaining healthy as could be desired, and the foot retained in place fully and perfectly.

At the end of *one week* from the operation upon the first foot, the other was treated in similar manner and with similar results. If, as sometimes occurred, the splints became too annoying, they were omitted for a few days, and the foot retained in place with adhesive plaster and bandages. The dressings were carefully watched and re-applied about once a week. The result is not yet fully apparent, but the child appears to have about as good feet as other children. The prominence upon the outer side of the foot is not fully overcome; the concavity upon the inner side is not wholly removed, but the result is much better than is usually obtained, and the chief reasons which I shall give for this, are these: The early division of the tendons; the immediate dressing of the foot, and the complete retention of it in the proper position. The three points are of importance, and the omission of some one of these conditions is the cause of common failure.

Early division of the contracted tendons is not always possible, since this depends upon circumstances which the surgeon cannot control, but the other two are of much more vital importance, and are altogether obtainable.

This is a representative case, and reported not on account of any peculiarity either of operation or treatment, but for the purpose of comparing actual results with theory, since the almost universal practice, is, to delay operation until the child is at least two months old, for fear of the effects upon the young infant; also to divide the tendons and allow the foot to remain undressed for a few days; and at last and more objectionable than all else, to apply somebody's boot, or splint, with the expectation of retaining the foot in proper position.

The results of this case will by no means be regarded as establishing the propriety of any particular plan of treatment in club foot, and it is only hoped to draw attention to the safety and propriety of early operation when opportunity offers, and also of immediate dressing, in proper position with suitable, efficient, yet simple means.

To depend upon splints, attached to boots, or otherwise arranged, to be applied by the mother or nurse, is believed to be a great mistake, and invariably followed by disappointment. Adhesive plaster, properly applied, is the most reliable means we possess of rotating and holding the foot in position. It is probably best and necessary, to use retentive dressings for at least one year in such cases, giving time for the bones to conform to the new condition in which they are placed, and the ligaments and tendons, time to unite and regain their functions.

